# OA24 Extended oligoarthritis with proximal muscle weakness and antinuclear antibody positivity associated with a novel genetic variant of uncertain significance in lipopolysaccharide-responsive and beige-like anchor protein (LRBA) gene

**DOI:** 10.1093/rap/rkae117.024

**Published:** 2024-11-05

**Authors:** Sandhya Govindarajan, Peter Arkwright, Alice Chieng

**Affiliations:** Manchester Foundation Trust, Manchester, United Kingdom; Manchester Foundation Trust, Manchester, United Kingdom; Manchester Foundation Trust, Manchester, United Kingdom

## Abstract

**Introduction:**

LRBA deficiency is associated with recurrent infections and immune dysregulation, particularly autoimmune cytopenias, enteropathy and intestitial lung disease, and in a few cases chronic polyarthritis and uveitis.

**Case description:**

A girl presented at three years old with history of not walking for two months with no history of trauma or fever. Examination revealed arthritis in her ankles and knees. Ultrasound showed synovial thickening in both knees and small effusion to hips. There was no evidence of uveitis at presentation. Her bloods showed raised C-Reactive Protein (CRP) 33 mg/L and Erythrocyte Sedimentation rate (ESR) 45 mm/h. Anti-nuclear and anti-centromere antibodies were positive. Diagnosis was made of oligoarticular JIA and she was treated with intra-articular steroids in both knees and ankles and was started on oral prednisolone, weekly oral methotrexate. On subsequent clinic review, she had started to walk with mild restriction to internal rotation of hips and difficulty in getting up from sitting. She was started on regular hydrotherapy, physiotherapy and occupational therapy. From five to 11 years old, she had four flares of uveitis, treated with prednisolone eye drops; and two flares of arthritis, treated with steroids. Her methotrexate was changed to subcutaneous and at eight years of age she was also started on subcutaneous adalimumab fortnightly. Due to prolonged proximal muscle weakness, abnormal gait and chronic pain in leg joints without clinical or radiological evidence of synovitis during clinic reviews, she underwent thorough neurological work up which was inconclusive for myopathies. Her creatinine kinase levels, thyroid profile, MRI brain and spine were normal. Genetic work up showed two variants in the LRBA gene, one pathogenic and the other was variant of uncertain significance (VUS). Her CTLA4 expression was normal by flow cytometry.

**Discussion:**

Three case reports have previously associated homozygous LRBA deficiency with polyarthritis. Compound heterozygous LRBA deficiency may present with various phenotypes including autoimmune disease affecting multiple organs, immune cytopenia and immune dysregulations including CTLA-4 expression. This report potentially widens the clinical spectrum of LRBA deficiency and given that it is a novel variant, prompts us to screen for this gene mutation in patients with JIA where remission is challenging and there is a need for continuous immunosuppressive or immune-modulatory therapy. Three case reports have previously associated homozygous LRBA deficiency with polyarthritis. Compound heterozygous LRBA deficiency may present with various phenotypes including autoimmune disease affecting multiple organs, immune cytopenia and immune dysregulations including CTLA-4 expression. This report potentially widens the clinical spectrum of LRBA deficiency and given that it is a novel variant, prompts us to screen for this gene mutation in patients with JIA where remission is challenging and there is a need for continuous immunosuppressive or immune-modulatory therapy. Three case reports have previously associated homozygous LRBA deficiency with polyarthritis. Compound heterozygous LRBA deficiency may present with various phenotypes including autoimmune disease affecting multiple organs, immune cytopenia and immune dysregulations including CTLA-4 expression. This report potentially widens the clinical spectrum of LRBA deficiency and given that it is a novel variant, prompts us to screen for this gene mutation in patients with JIA where remission is challenging and there is a need for continuous immunosuppressive or immune-modulatory therapy.

**Key learning points:**

• There is no single diagnostic investigation for JIA as the aetiology is complex and there are currently no specific clinical guidelines of when to consider genetic testing in patients with JIA. With the mainstreaming of genetic services and improved access to cheaper, advanced next-generation sequencing, we should consider looking into monogenic conditions that mimic JIA.

• The case illustrates the challenges of determining whether VUS are pathogenic in causing arthritis, or just incidental findings. Compound heterozygous LRBA may present within a spectrum of clinical features, further analysis with CTLA4 expression by flow cytometry and genetic analysis of siblings and parents will be helpful.

• Compound heterozygous LRBA variation has different clinical manifestation which needs personalised treatment. Treatment with abatacept (a soluble CTLA4 immunoglobulin fusion protein) which is reported in a case series of LRBA deficiency. Risks of continuous immunosuppressive treatment need to be balanced with more definitive hematopoietic stem cell transplantation (HSCT) previously used to achieved complete remission in LRBA deficiency.

